# The Design of a Computer Vision Sensor Based on a Low-Power Edge Detection Circuit

**DOI:** 10.3390/s25103219

**Published:** 2025-05-20

**Authors:** Suhyeon Lee, Yu Chan Yun, Seung Min Heu, Kyu Hyun Lee, Seung Joon Lee, Kyungmin Lee, Jiin Moon, Hyuna Lim, Taeun Jang, Minkyu Song, Soo Youn Kim

**Affiliations:** Department of System Semiconductor, Dongguk University, Seoul 04620, Republic of Korea; suhyeon.lee@dgu.ac.kr (S.L.); yunyu321@dgu.ac.kr (Y.C.Y.); michaelhea@dgu.ac.kr (S.M.H.); dice99@dgu.ac.kr (K.H.L.); le6124@dgu.ac.kr (S.J.L.); sgsgk1009@dgu.ac.kr (K.L.); sarah1008@dgu.ac.kr (J.M.); 2020110532@dgu.ac.kr (H.L.); tejang138@dgu.ac.kr (T.J.); mksong@dgu.ac.kr (M.S.)

**Keywords:** CMOS image sensor, computer vision, edge detection, low power consumption

## Abstract

We propose a complementary metal-oxide-semiconductor (CMOS) image sensor (CIS) that performs edge mask computation and detection during the analog-to-digital (A/D) conversion process to output 1-bit edge images. By utilizing the characteristics of the edge that can obtain a 1-bit image, the edge mask and thresholding operations are performed simultaneously during the A/D conversion process, thereby reducing memory capacity along with a high number of frames per second (FPS). Additionally, by implementing a 1-bit analog-to-digital converter (ADC) instead of a high-resolution ADC and counter through the 1-bit edge data obtained from the edge mask operation, both static and dynamic power consumption are reduced. The proposed CIS, fabricated with a one-poly six-metal CIS process with a 4T-active pixel sensor, has a core area of 2.546 mm × 1.923 mm in a chip area of 2.558 mm × 4.3 mm. The total power consumption is 1.52 mW at 23 FPS, with power supplies of 2.8 V and 1.5 V for the analog domain and 1.5 V for the digital domain.

## 1. Introduction

In computer vision, edge detection is a fundamental technique that allows for the extraction of critical features from images, facilitating accurate image segmentation by separating objects from the background. Based on these advantages, several studies have been conducted to apply computer vision techniques, such as biometric recognition and lane detection, to various applications [1,2,3]. In applications such as mobile face/iris recognition and autonomous driving lane detection, low power consumption (≤1 mW) [4] and high speed (≥30 FPS) are crucial requirements due to limited battery capacity and real-time object inference.

**Figure 1:**
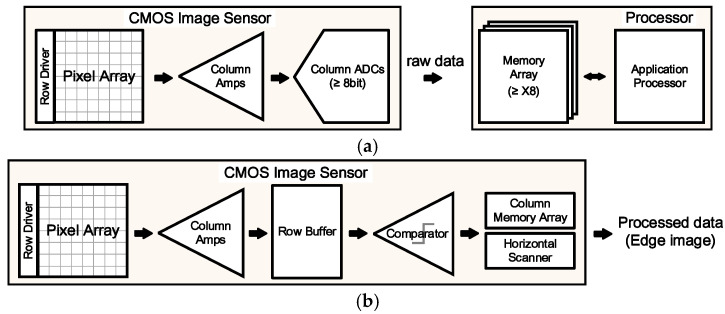
Image processing flow of (**a**) a conventional system and (**b**) the proposed system.

Conventional methods for object recognition, as illustrated in Figure 1, involve the following steps. The light incident on the photodiodes of a CMOS image sensor is converted into voltage values corresponding to its intensity through a voltage buffer. These voltage values are then converted into digital codes using an analog-to-digital converter (ADC), representing raw data, and are stored in a separate memory. Subsequently, edge detection or image classification operations are performed using the arithmetic logic unit (ALU) within the central processing unit or graphics processing unit of a mobile device’s application processor (AP) [5]. The energy consumed during data transfer from memory to the ALU significantly affects the overall power efficiency of computational implementation.

Therefore, research efforts have been dedicated to implementing edge detection and neural network operations within the readout circuit of a CMOS image sensor (CIS), leveraging the CIS for tasks beyond obtaining image information [6,7,8,9,10,11]. By integrating computations previously performed on external APs into the CIS, advantages can be gained in terms of power consumption and speed. However, the use of high-resolution ADCs with more than 8 bits is crucial during the A/D conversion process. High-resolution ADCs and counters account for approximately 40% of the total power consumption of the CIS [12], making them unsuitable for low-power CIS designs and resulting in a decrease in speed. Furthermore, since edge images are binary (1-bit), they can store only the essential features of an image using minimal memory capacity, which is a significant advantage. However, this advantage cannot be fully utilized when employing high-resolution ADCs.

In this study, we propose the implementation of a low-power, high-speed edge detection algorithm by simultaneously applying edge masks and performing detection using switch MUX and a comparator, as illustrated in Figure 1b. We directly output the edge image using a row buffer and 1-bit comparator, eliminating the need for high-resolution ADCs and counters. Furthermore, we utilize the output results of the edge image as inputs into a binarized neural network, which achieves image classification with low power consumption and reduced memory usage. This approach enables the implementation of low-power computer vision sensors.

This paper is structured as follows. Section 2 discusses the proposed low-power edge detection circuit, including circuit design and implementation. Section 3 describes the fabricated chip and the measurement environment, followed by the analysis of measurement results using various evaluation metrics. The conclusion is presented in Section 4.

## 2. The Proposed CIS for Low-Power Edge Detection

### 2.1. Architecture Flow

**Figure 2:**
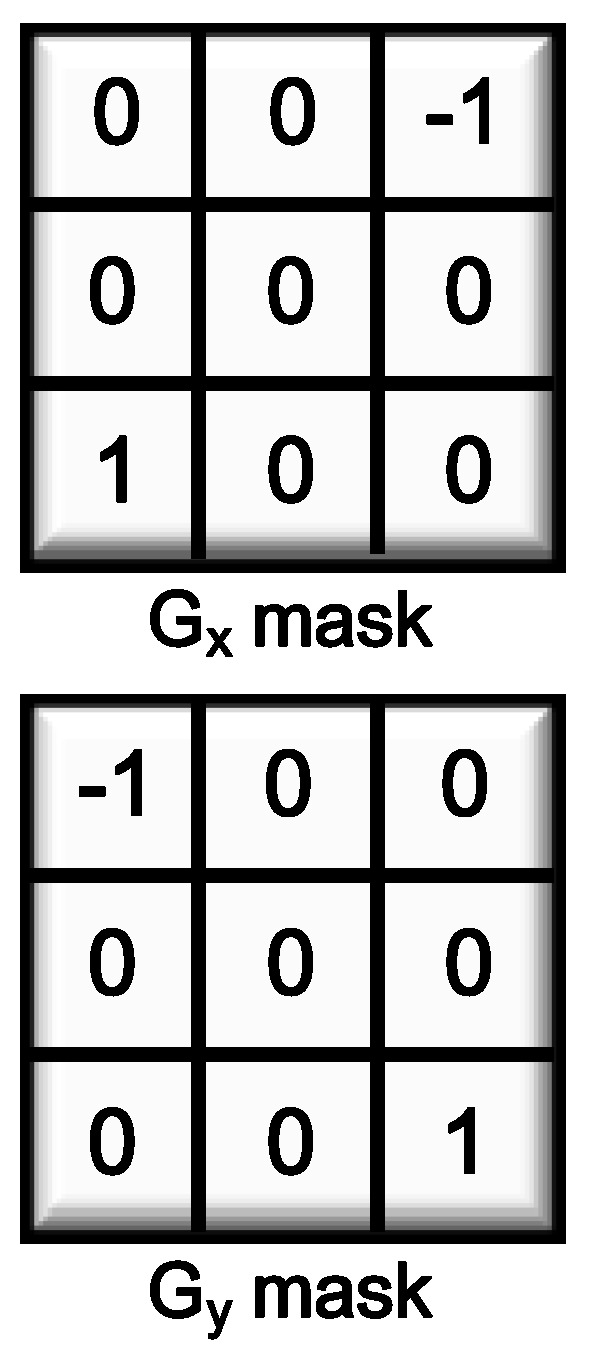
Built-in mask.

**Figure 3:**
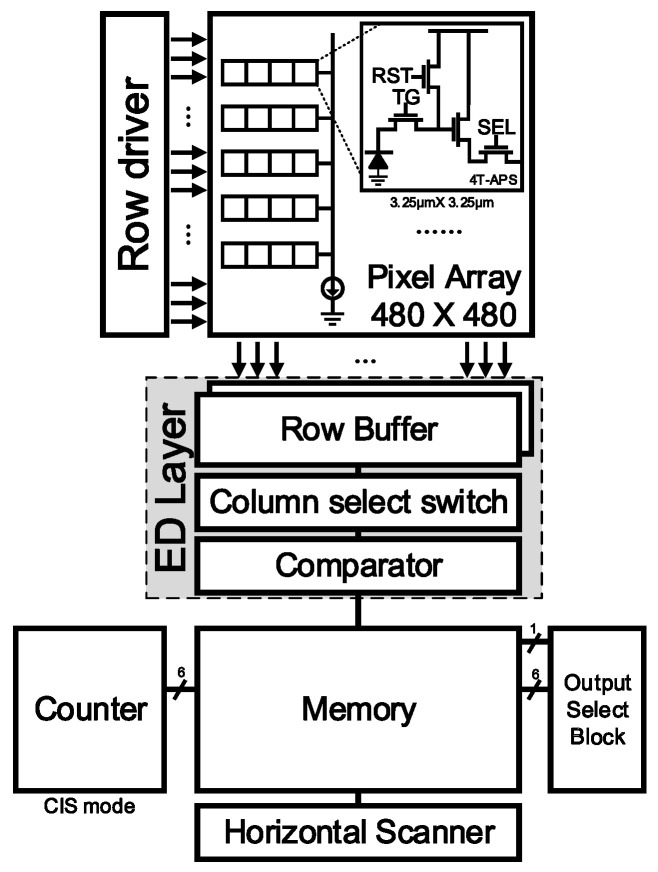
The proposed CVS Architecture.

The built-in edge mask [9] in the presented CIS structure is illustrated in Figure 2. This mask demonstrates performance comparable to the widely used Sobel mask, achieving a Pratt’s figure of merit result of 97.24% and offering ease of hardware implementation, as well as efficient detection of diagonal edges. The overall block diagram of the proposed low-power edge detection CIS system is illustrated in Figure 3. The system comprises a 480 × 480 pixel array consisting of 4T-active pixel sensors (APS). To facilitate the capacitor and column amplifier layout in the rear block, four pixels are grouped together as a unit, with only one pixel utilizing a voltage buffer. As a result, an active 120 × 120 pixel array is obtained. It should be noted that the image resolution required in vision-based sensing is mainly determined by the object size, the distance between the sensor and the object, and the camera’s field of view. Dimensions of 120 × 120 pixels can detect the presence of an object of 10 mm in size at a distance of about 1 m [13]. The implementation of the built-in mask involves a row buffer block that stores two rows of data. During the pixel readout process, correlated double sampling (CDS) is performed to eliminate noise and preserve the sampled values. Additionally, an edge detection (ED) layer is constructed, comprising a column select switch (CSS) block for striding the mask by one and a comparator block for applying a threshold to the computed mask values, resulting in the generation of the final edge output. The detected edge data are stored in a 1-bit column memory and sequentially read out using a horizontal scanner.

**Figure 4:**
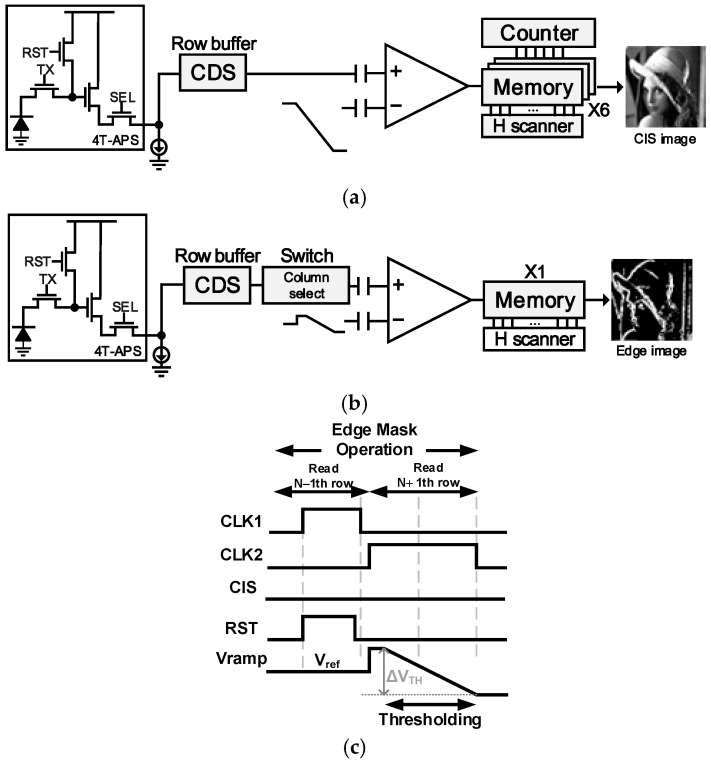
System flow with different modes: (**a**) CIS mode and (**b**) edge detection mode, and (**c**) comparator circuit and edge detection operations.

The system operates in two modes, CIS mode and edge detection mode, as shown in Figure 3. In CIS mode (Figure 4a), the CIS image is outputted by directly converting the voltage values that have undergone CDS into a multi-bit digital representation using a 6-bit single-slope analog-to-digital converter (SSADC), without the application of the edge mask. On the other hand, in edge detection mode (Figure 4b), the edge image is generated by applying the built-in mask to the two rows of data stored in the row buffer, followed by mask computation and threshold application using the CSS and comparator, respectively. The digital code values are outputted based on the selected mode using the output select block. Figure 4c shows the comparator and edge detection timing used in this study.

When the input voltage ramp is included in the threshold voltage range, a comparator’s out value that flips from High to Low is generated, and this signal is applied as a clock for the negative edge triggered Flip-Flop (N-ETDFF). VDD is input to the data of this N-ETDFF, and when triggered by the clock, Qb is output as Low, completing the thresholding process. In this way, unlike the existing method where the ramp had to be applied for a time equal to the clock frequency multiplied by the n-bit code, high fps can be implemented by simultaneously applying contour detection and thresholding without being limited by the ramp application time.

### 2.2. Pixel Voltage Sampling and Edge Mask Operation

**Figure 5:**
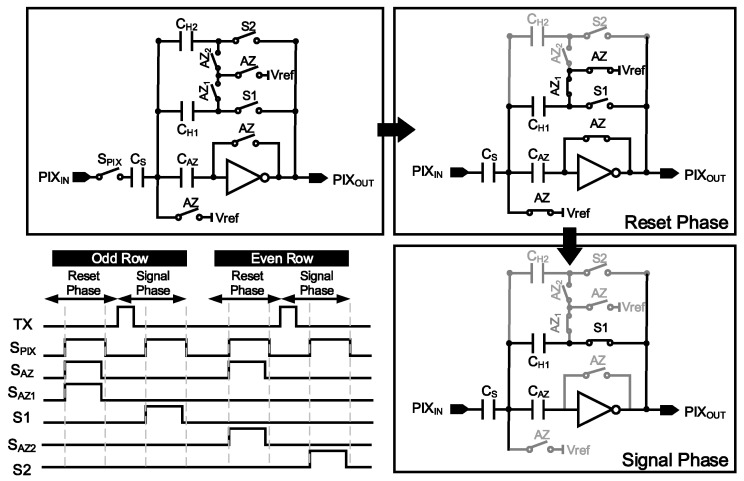
Row buffer operation according to the phase and timing diagram.

The voltage value converted from the voltage buffer of the pixel is stored in the holding capacitor through CDS in the row buffer [14]. Figure 5 illustrates the switched capacitor structure of the row buffer and the timing diagram and circuit operation according to the phases.

The V_rst_ in the reset phase and V_sig_ in the signal phase are input to PIX_IN_, and the charge conservation principle using the sampling capacitor, holding capacitor, and operational transconductance amplifier (OTA) is used to drive the PIX_OUT_ of Equation (1). In this circuit, as C_S_ and C_H_ have the same capacitance, it outputs V_ref_+ΔPIX, where 1V of V_ref_ is used in this paper.

(1) $$PIX_{OUT}=V_{ref}+\frac{C_{s}}{C_{H}}\left(V_{rst}-V_{sig}\right)=V_{ref}+∆PIX$$

**Figure 6:**
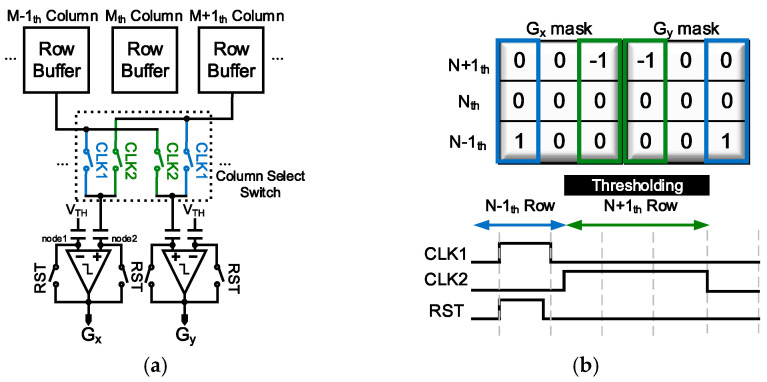
(**a**) Column select switch and comparator in the edge detection layer. (**b**) Timing diagram for mask operation.

The two holding capacitors store odd-row and even-row data, respectively, and the values required for edge mask operations are transferred to the back-end circuitry, as shown in Figure 6a, through S1 and S2. The pixel values with the offset removed through CDS undergo built-in mask operations using the column select switch and comparator. Figure 6b represents a timing diagram for the mask operation of x-direction and y-direction gradient, G_x_ and G_y_, where the blue color corresponds to the data operation in the first row and the green color represents the data operation in the third row. With respect to G_x_, when CLK1 is activated, the data from the (N − 1)_th_ row of the (M − 1)_th_ column (=ΔPIX_(N−1,M−1)_) are transmitted to the comparator. At this moment, with RST turned ON, the charge quantity equal to the positive input capacitance of the comparator (=C_IN_) multiplied by V_LT_-ΔPIX1 is stored. Here, V_LT_ represents the logic threshold voltage obtained when the input and output of the first-stage circuit, which is a single-ended differential amplifier inside the comparator, are connected in a feedback loop. Then, RST is turned off, and CLK2 is activated to receive the data from the (M + 1)_th_ row of the (N + 1)_th_ column (=ΔPIX_(N+1,M+1)_). As a result, the charge quantity of the capacitor changes to C_IN_{V_LT_-(ΔPIX_(N−1,M−1)_-ΔPIX_(N+1,M+1)_)}, and the mask operation in the G_y_ direction is simultaneously performed in the same manner. This is expressed by Equation (2).

(2) $$G_{x}=V_{LT}-\left\{V_{ref}+∆PIX_{\left(N-1,M-1\right)}\right\}-\left\{V_{ref}+∆PIX_{\left(N+1,M+1\right)}\right\}\;\;\;\;\;\;\;\;=V_{LT}-\left\{∆PIX_{\left(N-1,M-1\right)}-∆PIX_{\left(N+1,M+1\right)}\right\}\;G_{y}=V_{LT}-\left\{V_{ref}+∆PIX_{\left(N-1,M+1\right)}\right\}-\left\{V_{ref}+∆PIX_{\left(N+1,M-1\right)}\right\}\;\;\;\;\;\;\;\;=V_{LT}-\{∆PIX_{\left(N-1,M+1\right)}-∆PIX_{\left(N+1,M-1\right)}\}$$

### 2.3. Thresholding Operation

**Figure 7:**
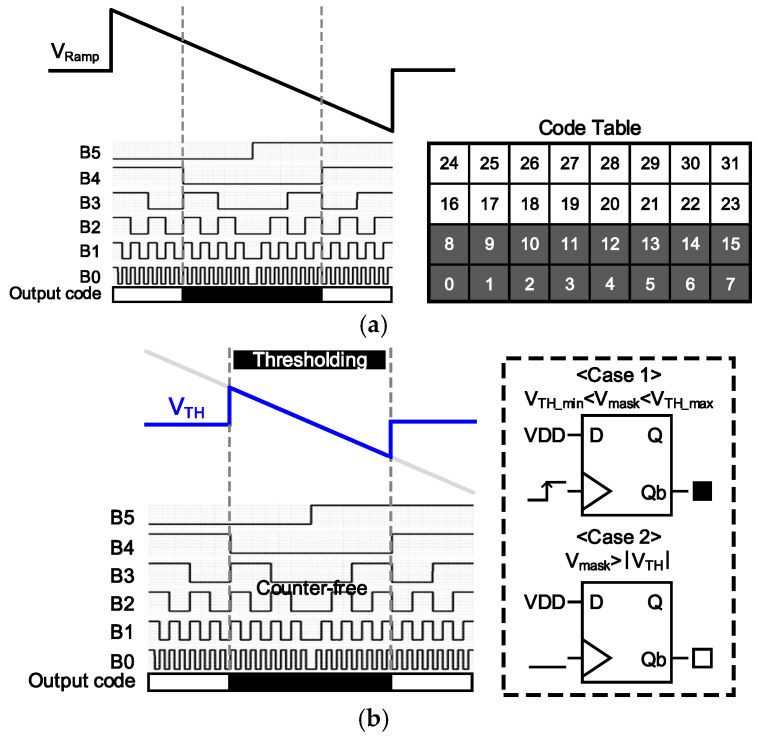
(**a**) Conventional and (**b**) proposed thresholding method.

**Figure 8:**
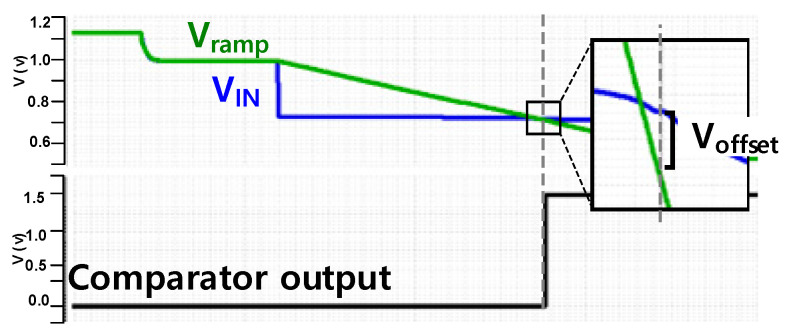
The offset voltage generated during the comparison operation.

After the mask operation is completed, the voltage values may contain noise or a false edge. Therefore, it is necessary to apply a thresholding process that treats only values above a certain threshold as edges. This process requires separate software processing [9]. In this circuit, we propose an edge detection circuit structure that combines edge mask operations and threshold application processes simultaneously to achieve a high processing speed and reduce unnecessary counter toggling in order to scale down the power supply of the comparator for a low-power edge detection circuit. Equation (2) for the completed mask operation can have a positive or negative value based on the magnitudes of ΔPIX_(N-1,M-1)_ and ΔPIX_(N+1,M+1)_. Therefore, to convert this into digital code, a ramp waveform is applied as $V_{LT}\pm ∆V_{PP,PIXEL}$ (i.e., dynamic range of pixels) and an additional counter is employed to generate a clock signal that increases progressively from the edges towards the center based on the V_LT_. For example, as shown in Figure 7a, when applying a threshold of 0.5 to the converted full code in order to highlight the half portion, the 32 codes of the 5-bit data (excluding the most significant bit, representing polarity) will output as 0 for the lowest 16 codes and 1 for the upper 16 codes. We apply this method by using the SSADC operation to apply a threshold using a ramp signal during the A/D conversion process so that a separate process is not required after high-resolution image conversion. As an example with Th = 0.5, as shown in Figure 7b, a ramp slope is applied only to the window that outputs 0, which is the edge, among 1LSBХ16, and when the mask operation value is included within this range, the flip signal is connected to the CLK of TGFF, and when the data value being forced by VDD is triggered, the Qb signal that is output is used to determine that it is not edge data. Conversely, if the operation value is not included within the threshold window, the Qb signal remains unchanged and outputs High, indicating that it is edge data. This approach, as the ramp input range is not limited by the maximum voltage range of the pixel, allows for scaling down the supply voltage of the comparator from 3.3 V to 1.5 V. Moreover, since there is no need for a counter, both static and dynamic power consumption can be reduced. Furthermore, unlike previously, where the ramp time was set to be equal to the clock frequency multiplied by 2^n^, where n represents ADC resolution, in this method, by performing edge detection and threshold application simultaneously, the frame rate can be improved without being limited by the input time. The detected G_x_ and G_y_ mask results are combined by an OR gate and output as 1-bit data. See Figure 8.

Table 1 summarizes the post-simulation results of offset voltage, gain, and unity gain frequency for different corners. In all process corners, the values of V_offset_ are lower than the 1LSB (=15.6 mV) threshold, which is based on a 5-bit resolution with a ramp V_pp_ of 500 mV. This indicates that the comparator achieves an accuracy comparable to a 5-bit ADC.

## 3. Experimental Results

### 3.1. Layout and Chip Photograph of the Proposed CIS

**Figure 9:**
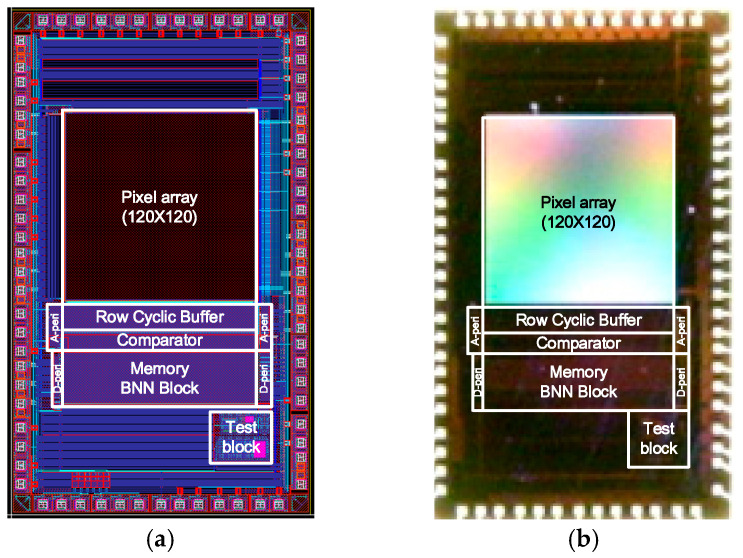
(**a**) Layout of the proposed CIS and (**b**) mircrophotograph of the fabricated chip.

Figure 9a,b present the layout and a photograph, respectively, of the proposed CIS. The chip was fabricated using a 0.11 μm one-poly six-metal CIS process, with a total area of 2.558 mm (H) × 4.300 mm (V). To lay out the proposed structure in an in-column method within a 480 × 480 pixel array, four pixels are grouped as a unit, with 120 × 120 pixels actively utilized. The pitch of each pixel is 3.25 × 3.25. In the analog domain, the pixel and row buffer operate at a supply voltage of 2.8 V, while the comparator operates at a voltage of 1.5 V. In the digital domain, the memory and horizontal scanner operate with a power supply voltage of 1.5 V.

### 3.2. Measurement Results

**Figure 10:**
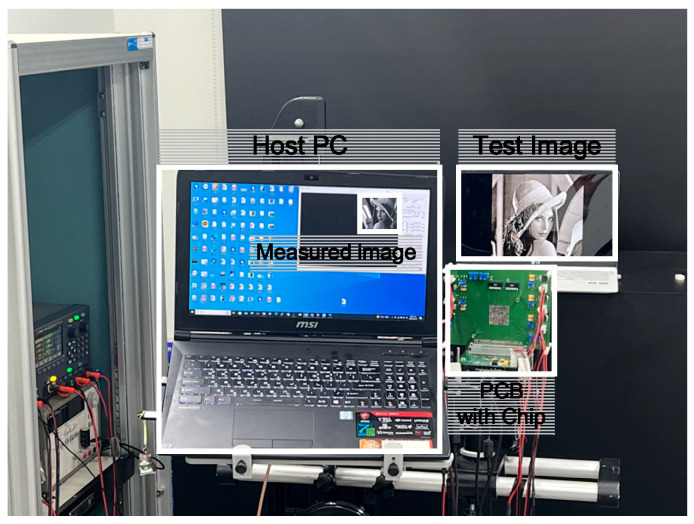
Measurement environment.

Figure 10 shows the environment for measuring the fabricated chip. The measurement process involves coding the necessary signals for the internal circuit operation of the chip in Verilog, which are then applied to the FPGA connected to the motherboard from the host PC. These signals are then applied to the chip on the daughterboard, and the measured test image is observed using an image program.

**Figure 11:**
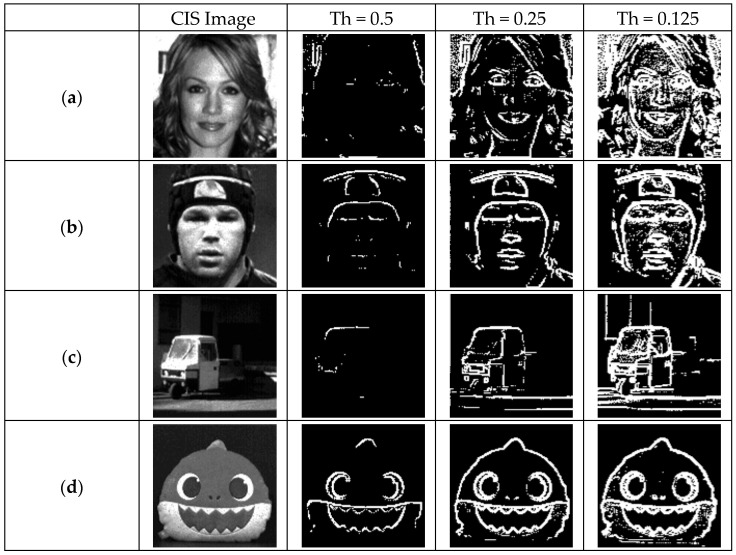
Measured edge images using 3 threshold values. (**a**) Woman’s face; (**b**) man’s face; (**c**) car; (**d**) shark doll.

Figure 11 showcases images corresponding to three different thresholds. (a) and (b) represent results obtained from measuring human faces, while (c) and (d) depict measurements of objects. The thresholds 0.5, 0.25, and 0.125 refer to the applied ramp slopes of 16 LSB, 8 LSB, and 4 LSB, respectively, based on the ramp V_pp_ used for the 6-bit CIS image measurement. As the threshold decreases, the number of edge outputs increases, resulting in corresponding changes in the images.

**Figure 12:**
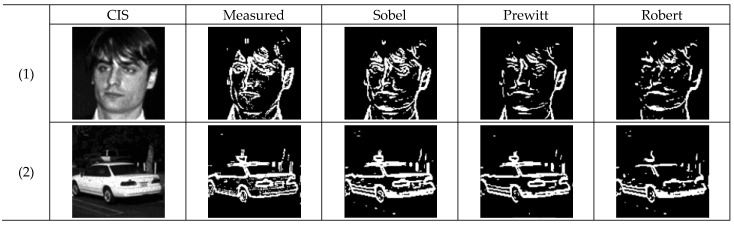
Meaured image using the built-in, Sobel, Prewitt and Robert masks.

The measured images, shown in Figure 12, are compared to the Sobel, Prewitt, and Robert masks based on the reference measurement image. The evaluation involves comparing the mean squared error (MSE), peak signal-to-noise ratio (PSNR) [15], and accuracy, which represent image similarity.

MSE quantifies the average squared difference between the original and reconstructed images. A lower value indicates a higher similarity between the images. PSNR is calculated by logarithmically transforming the MSE, as described in Equation (3), where R represents the maximum possible value of the input image, typically 255 for an 8-bit image. PSNR is indicated in terms of dB, and a higher value signifies greater similarity between the images. Generally, a PSNR value above 30 dB is considered indicative of high quality.

(3) $$PSNR[dB]=10\log_{10}\left(\frac{R^{2}}{MSE}\right)$$

Table 2 and Table 3 summarize the MSE and PSNR values by comparing the images obtained by applying the three masks to the measured edge images. Both Image (1) and Image (2) have PSNR values above 55, demonstrating performance comparable to the Prewitt mask. Table 4 calculates accuracy by determining the ratio of pixels indicating the same code among the total number of pixels when overlaying the measured image with the images obtained using the three masks, as defined in Equation (4). It can be observed that, excluding the Robert mask, which is sensitive to noise, both the Sobel and Prewitt masks exhibit an accuracy of over 90%.

(4) $$Accuracy[\%]=\frac{Number\;of\;pixels\;with\;matching\;code}{Total\;number\;of\;pixels}\times 100$$

Table 5 summarizes the main performance metrics of the proposed CIS with edge detection, while Table 6 presents a performance comparison of edge detection algorithms integrated into edge detection with previous studies [5,7,9]. All of these studies [5,7,9] utilize ADCs with a resolution of 8 bits or higher. References [5,7] convert pixel data into high-resolution CIS images in the digital domain for edge detection, whereas [9] converts analog edge information into 8-bit digital codes and applies thresholding through additional software processes to obtain the final edge information. In this study, we only use a low pixel resolution of 120 × 120 and consume significantly reduced levels of power, up to 83.8% lower compared to other papers, by detecting edges without using counters alongside a low supply voltage ADC. In the process of first checking pixel operation for edge detection sensor mode, additional power consumption was added to circuits such as the memory array and ADC. Therefore, additional power optimization is possible in the process of supporting both CIS and edge detection modes. The proposed circuit can output a 1-bit outline image directly without an n-bit image conversion process by limiting the ramp voltage itself to the threshold voltage. As a result, the continuous clock toggling required for counter operation is unnecessary, resulting in a reduction of dynamic power consumption of approximately 96.6% from 2.91 μW of dynamic consumption consumed in the method of [9] to 98.74 nW during A/D conversion. Additionally, by performing edge operations and thresholding simultaneously during the A/D conversion process, we can achieve a high frame rate. The thresholding process can be applied to all types of mask operations, and by extracting only important information as edges from images, it can be utilized as a preprocessing step for image classification.

## 4. Conclusions

In this paper, we demonstrate an edge mask calculation and detection CIS using a 1-bit ADC fabricated with a 1P6M 0.11 µm CIS process. Instead of using a high-resolution ADC, we implement an edge detection algorithm in the readout circuit of the CIS using an in-column approach, allowing us to output edge images without the need for additional processing. We employ an SSADC structure, which is suitable for column-parallel implementation. By applying a ramp signal only to the regions that do not require edge detection, we avoid converting the entire dynamic range of pixels into digital codes and directly output 1-bit images. This approach reduces unnecessary counter toggling and decreases power consumption. As a result, in edge detection mode, our proposed CIS consumes 1.52 mW of power while achieving a speed of 235 frames per second (FPS), satisfying the criteria of low power consumption and a fast inference speed of over 30 FPS in battery-constrained small-form factor devices. We believe that the proposed CIS for edge detection can be applied to low-power computer vision sensors for the rapid detection of objects or faces.

## Figures and Tables

**Table 1:** Post-simulation results of the comparator under process variation.

Process	V_offset_ [mV]	Open Loop Gain [dB]	Unity Gain Frequency [MHz]
ff	6.39	71.3	59.3
nn	8.50	72.2	46.1
ss	11.4	72.1	35.9

**Table 2:** MSE results of 3 mask images based on the measured image.

MSE	(1)	(2)
Sobel	0.14	0.12
Prewitt	0.11	0.10
Robert	0.15	0.12

**Table 3:** PSNR results of 3 mask images based on the measured image.

PSNR [dB]	(1)	(2)
Sobel	56.66	57.17
Prewitt	57.65	58.16
Robert	56.38	57.35

**Table 4:** Accuracy results of 3 mask images based on the measured image.

Accuracy [%]	(1)	(2)
Sobel	90%	92%
Prewitt	90%	93%
Robert	86%	87%

**Table 5:** Performance table of the proposed CIS with edge detection.

Process	0.11 μm 1P6M CIS process
Pixel size	3.25 μm × 3.25 μm
Pixel type	4T-APS
Pixel resolution	120 × 120
Chip area	2.558 mm × 4.3 mm
Core area	2.546 mm × 1.923 mm
Supply voltages	2.8V,1.5V(Analog)/1.5V(Digital)
ADC resolution	1bit (5-bit ADC-comparable accuracy)
Clock frequency	10 MHz
Power consumption	1.52 mW
FPS	235 @ Edge detection mode 220 @ CIS mode

**Table 6:** Performance comparison table for CIS integrated edge detection.

	[5]	[7]	[9]	This work
Edge Image	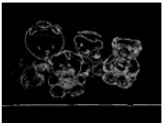	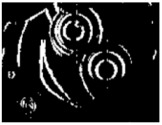	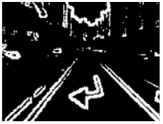	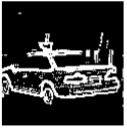
Progress	0.09 μm 1P4M CIS	0.18 μm 1P4M CIS	0.11 μm 1P4M CIS	0.11 μm 1P6M CIS
Resolution	1920 × 1440	160 × 120	160 × 120	120 × 120
Pixel pitch	1.4 μm × 1.4 μm	4.9 μm × 4.9 μm	3.2 μm × 3.2 μm	3.25 μm × 3.25 μm
Supply voltage	2.8 V (Pixel)/3.3 V (Analog)/1.2 V (Digital)	2.8 V (Pixel)/1.8 V (Circuit)	3.3 V (Pixel, Analog) 1.5 V (Digital)	3.3 V (Pixel)/2.8 V (Analog)/1.5 V (Analog, Digital)
Power	9.4 mW	4.3 mW	9.4 mW	1.52 mW
FPS	60	3200	145	235
* FoM [pJ/pixel/frame]	56.7	70	3376.4	** 449.2

* FoM = Power/(N_PIX_ × FPS); ** FoM = 28.1 [pJ/pixel/frame] for 480 × 480 array with 58.75 FPS.

## Data Availability

The datasets generated in this study are available from the corresponding author upon reasonable request.
